# Diagnosing Systemic Mastocytosis: State of the Art

**DOI:** 10.1111/ijlh.70011

**Published:** 2025-10-07

**Authors:** Anton Rets, Tracy I. George

**Affiliations:** ^1^ Department of Pathology University of Utah Salt Lake City Utah USA; ^2^ ARUP Laboratories Salt Lake City Utah USA

**Keywords:** bone marrow, mast‐cell disease, mastocytosis, neoplasia, pathology

## Abstract

With the advent of effective multikinase and selective tyrosine kinase inhibitors in systemic mastocytosis, diagnosing this rare disease has been critical to improving patient morbidity and mortality. This state‐of‐the‐art review interprets the international diagnostic criteria, including differences between the WHO 5th edition classification and the International Consensus Classification. Subclassification of systemic mastocytosis is critical for correct therapeutic strategies, and diagnostic difficulties are described for the practicing pathologist. Morphologic mimics, which require alternative treatment, are discussed.

## 1 Introduction

The last decade has seen the introduction of multikinase and selective tyrosine kinase inhibitors for the treatment of systemic mastocytosis (SM), a rare disease typically characterized by the *KIT* p.D816V mutation in more than 95% of patients [1, 2, 3, 4]. These therapeutic successes have driven the need for accurate diagnosis and subclassification of SM, a disease most physicians may never see during their careers. Driven by the longstanding European Competence Network in Mastocytosis (ECNM), diagnostic criteria for SM were first developed in 2000 and published in 2001 [5]. Subsequent revisions have been based on these original guidelines, with the most recent adapted by the World Health Organization (WHO) 5th edition classification and the International Consensus Classification (ICC) in 2022 [6, 7, 8, 9, 10]. In recognition of the difficulties of diagnosing a disease infrequently encountered by physicians, substantial educational efforts have been undertaken by international scientific societies, physician specialty societies, with international conferences and numerous publications. Indeed, approximately 40% of patients enrolled in a pivotal clinical trial for advanced SM were found to have been initially misclassified after review by a central adjudication committee including expert pathology review [manuscript in preparation]. Further difficulties include the long time to diagnosis after initial symptoms, with a median of 7 years from symptom onset to diagnosis of SM [11]. This review addresses our latest understanding of the application of diagnostic criteria in clinical practice, including diagnostic difficulties with the pathology of mastocytosis. The recognition that mastocytosis may be “hiding” in certain myeloid malignancies has changed the diagnostic algorithm for this disease. Another example of a diagnostic algorithm is that adults who present with cutaneous manifestations of mastocytosis typically have evidence of systemic disease. Morphologic mimics of SM are also reviewed, as the genetic drivers of these mimics require alternative therapies.

## 2 Diagnostic Criteria

Updates to the diagnostic criteria and classification of SM have been published by the WHO in 2022 [6, 7], while the Clinical Advisory Committee endorsed by the Society for Haematopathology and the European Association for Haematopathology International Consensus resulted in the ICC, also in 2022 [8, 9, 10]. The overall diagnostic criteria are quite similar between the two classification systems, with only a few differences discussed below.

### 2.1 Major Diagnostic Criterion

**FIGURE 1:**
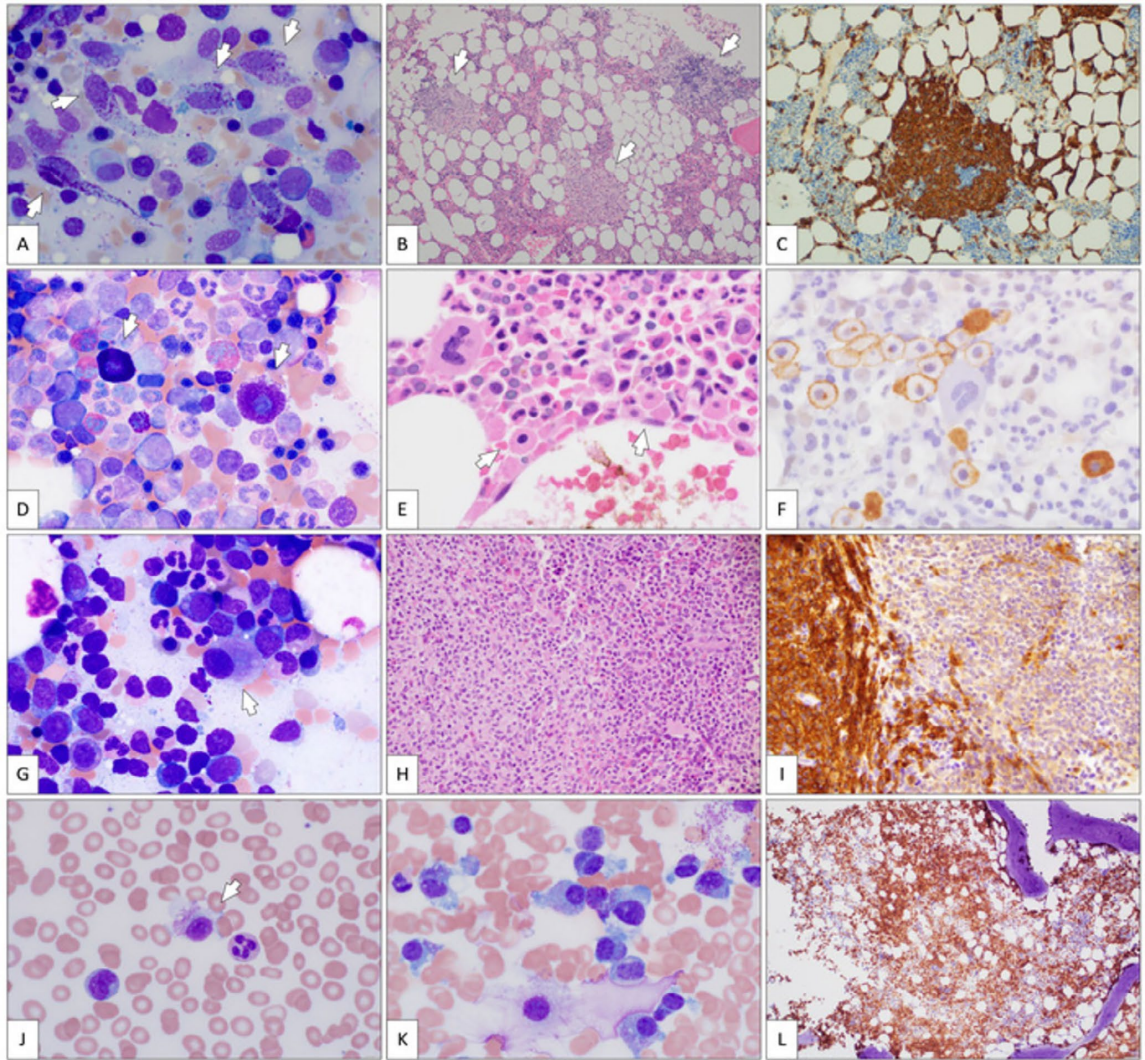
Key morphologic findings in different subtypes of systemic mastocytosis. (A–C) Indolent systemic mastocytosis. The aspirate smear (A) contains numerous mast cells (arrows), most of which are atypical (spindle‐shaped). Decreased number of cytoplasmic granules may make their recognition challenging (Giemsa stain, 100×). The bone marrow core (B) contains multifocal large dense aggregates of mast cells (arrows) (hematoxylin and eosin, 10×). The mast cell aggregates are highlighted by strong CD117 staining (anti‐CD117 immunostain, 20×) (C). (D–F) Well‐differentiated indolent systemic mastocytosis. The neoplastic mast cells (arrows) in marrow aspirate smear (D) show mature features, for example, round/oval shape and round centrally placed nuclei with mature chromatin. They, however, are larger than normal mast cells and contain coarser cytoplasmic granules (Giemsa stain, 100×). (E) In the core section. (E), the mast cells form loose clusters of less than 15 cells (arrows) (hematoxylin and eosin, 20×). These loose clusters are highlighted by CD117 (anti‐CD117 immunostain, 10×) (F). (G–I) Systemic mastocytosis with associated hematological neoplasm (chronic myelomonocytic leukemia). The marrow aspirate smear (G) from a patient with cytopenias and monocytosis shows myelomonocytic predominance and dysplastic megakaryocytes (arrow) but no mast cells (Giemsa stain, 100×). The core biopsy (H) contains large dense aggregates of mast cells (the left area of the image). The remaining marrow shows myelomonocytic hyperplasia (hematoxylin and eosin, 10×). (C) The mast cell aggregate is highlighted by CD117 (anti‐CD117 immunostain, 10×) (I). (J–L) Mast cell leukemia. A circulating mast cell in the peripheral blood (arrow) (J). The degree of atypia can make the recognition of mast cells challenging (Giemsa stain, 100×). Numerous morphologically atypical mast cells (promastocytes) in the marrow aspirate smear (K), comprising > 20% (Giemsa stain, 100×). CD117 immunostain (L) highlights diffuse interstitial mast cell infiltrates effacing normal marrow architecture (anti‐CD117 immunostain, 10×).

Conceptually, the diagnosis of SM requires the detection of an abnormal proliferation of mast cells (MCs) in extracutaneous organs (the major diagnostic criterion) and demonstration of their clonal nature (minor diagnostic criteria) (see Table 1). Morphologic examination of the affected organ(s), particularly bone marrow, is an essential component of the diagnostic workup [5]. To meet the major diagnostic criterion, one must demonstrate “multifocal dense aggregates of MCs in the bone marrow (BM) and/or other extracutaneous organs” [5]. A dense aggregate is defined as 15 or more MCs in close proximity [5]. MCs can be very difficult to recognize on a hematoxylin and eosin‐stained tissue section where they often appear as intermediate‐sized cells with round to oval “bland” nuclei (i.e., smooth nuclear border, dense chromatin devoid of nucleoli), and moderately abundant pink cytoplasm with only occasionally identifiable granularity (Figure 1) [12]. These morphologic features may lead to confusion of MCs with histiocytes and even fibroblasts, especially when the MCs are spindle‐shaped and are associated with fibrosis [12]. Easily recognizable dense aggregates of MCs may be interpreted as granulomata, compromising the diagnostic workup. MC aggregates are typically associated with mature lymphocytes, often forming lymphoid aggregates admixed with the MCs and/or an increased number of eosinophils [12]. Focal fibrosis is also a common feature of the MC aggregates comprised of reticulin and collagen fibrosis [12]. Thus, the presence of aggregates of epithelioid or histiocyte‐like appearing cells in association with lymphoid aggregates, increased eosinophils, and/or fibrosis must raise a suspicion for an MC proliferation, and appropriate additional stains should be performed [12].

**TABLE 1:** Diagnostic criteria for systemic mastocytosis.

Major	Multifocal dense aggregates of mast cells
Minor	> 25% mast cells with atypical morphology
	Activating *KIT* mutation
	CD2, CD25 and/or CD30 expression on mast cells
	Serum total tryptase > 20 ng/mL

In the past, the identification of MCs in histologic sections was aided by special stains, such as Giemsa or toluidine blue. On aspirate smears, the MCs may be recognized by their ability to cause color shift of the cationic dyes. For example, when stained with toluidine blue, MCs are red‐purple (metachromasia) in a blue background (orthochromasia) [5]. Despite broad availability and the lower cost of special stains, immunohistochemistry provides significantly improved sensitivity, specificity, and ease of interpretation. The most sensitive marker for MCs is CD117 (c‐kit), a receptor tyrosine kinase expressed on the MC surface, essential for the survival of normal and clonal MCs. In addition, strong c‐kit expression by MCs makes anti‐CD117 one of the key immunostains to enumerate MCs and assess their distribution and cytomorphology (Figure 1). CD117 expression, however, is not specific to MCs as it highlights other hematopoietic cells, such as blasts, and immature myeloid and erythroid precursors, which often demonstrate variable stain intensity [13]. In other tissues, CD117 is expressed on melanocytes, interstitial cells of Cajal, endothelium, etc. [13]. To alleviate this pitfall, immunostain for tryptase must be included in the panel. Although tryptase is the most specific marker for MCs, some basophils may show weak positivity. Similarly, tryptase may also be expressed by myeloid neoplasms, although typically dim and variable; this is also associated with increased serum tryptase levels [14]. In these instances, however, the myeloid neoplasms show the morphologic features typical of acute myeloid leukemia and chronic myelomonocytic leukemia, for example. Downregulation of tryptase expression by the neoplastic MCs may result in their negativity by immunostaining. In SM involving the gastrointestinal tract, the MCs typically show dim to negative expression of tryptase [15]. Thus, a panel of immunohistochemical stains, typically including CD117, tryptase, CD25 (CD30 if CD25 is negative), and CD34, is recommended in the workup of potential mast cell neoplasms [16]. CD2 is best evaluated by flow cytometry (FC), although aberrant CD2 expression in SM can also be detected by immunohistochemistry [16].

### 2.2 Minor Diagnostic Criteria

The minor diagnostic criteria are MC atypia (morphologic and immunophenotypic), presence of an activating mutation in the *KIT* gene, and elevated basal serum tryptase (BST) levels [5].

Morphologic atypia of MCs is determined in well‐sampled bone marrow aspirate smears or touch preparations. If those are not available, bone marrow tissue sections may also be used. This diagnostic criterion requires that > 25% of MCs are spindle‐shaped or otherwise morphologically atypical (e.g., showing features of immaturity) [5]. In aspirate smears, MCs are most readily identified in proximity to the marrow particles. The most common atypia in SM is a spindled shape (Figure 1). Spindle‐shaped MCs show oval nuclei with mature chromatin devoid of nucleoli [12]. The cytoplasm may be less granular in comparison to well‐differentiated MCs, and the granules may exhibit irregular distribution in cells which contain elongated cytoplasmic extensions [12]. Immature MCs may be designated as “promastocytes” or “metachromatic blasts” [5]. The former demonstrate atypical nuclear shapes (i.e., reniform or bilobed), and less condensed chromatin (Figure 1); their cytoplasm contains metachromatic granules [12]. Metachromatic blasts are distinguished by their immature nuclear chromatin. They have round or oval nuclei, often with conspicuous nucleoli, increased nuclear‐cytoplasmic ratio, and metachromatic cytoplasmic granules, which may be few and coarse [12]. In rare cases, neoplastic MCs can be markedly enlarged and pleomorphic, resembling atypical megakaryocytes or bizarre cells [12]. In contrast, “well‐differentiated” MCs are characterized by round or ovoid shape, centrally placed round nucleus with mature chromatin devoid of nucleoli, and abundant cytoplasm containing numerous medium‐sized metachromatic granules (Figure 1) [17, 18]. These well‐differentiated MCs are typically enlarged in size compared to normal or reactive MCs.

Recognition of this morphology is critical as well‐differentiated SM (WDSM) has unique features, including distinct morphologic, immunophenotypic, and molecular characteristics with a clinical response to imatinib [17, 18]. WDSM typically shows diminished to negative expression of CD25 and bright CD30 expression [18]. Molecular characteristics include only a minor subset of patients with WDSM demonstrating the *KIT* p.D816V mutation, with many patients showing wild type or alternative *KIT* mutations [17, 18]. Clinically, these patients respond to imatinib therapy [17].

Immunophenotypic aberrancy is defined as the expression of CD2 and/or CD25 and/or CD30 [6, 7, 8, 9]. The immunophenotype can be assessed by immunohistochemistry and/or FC. Both methodologies complement each other and, if possible, both should be performed. FC allows multiparametric immunophenotypic characterization of MCs, including the assessment of all key markers of aberrancy. The main limitation of FC is the relatively low number of MCs in tested specimens, even in cases of SM. Therefore, optimization of FC evaluation, which includes a significant increase in the number of events (i.e., at least 1 million) and careful selection of marker‐fluorochrome combinations to alleviate the autofluorescence of MCs, is required to achieve clinically meaningful levels of sensitivity [19, 20]. The creation of synthetic abnormal MC particles will likely help in the development and standardization of FC of rare cells, such as MCs [21]. Immunohistochemistry allows the assessment of the morphologic patterns of marker expression, but only one marker can be evaluated in each section, as multiplex staining is not widely available in clinical practice. CD25 appears to be the most frequently expressed abnormal marker on neoplastic MCs in BM with bright membrane expression [22]. CD25 also highlights a subset of the T cells (T regulatory cells) and megakaryocytes, with the latter showing dim expression. Assessment of CD2 expression on MCs by immunohistochemistry can be difficult to assess since CD2 is a pan‐T cell marker highlighting all T cells in the marrow, but it typically shows dimmer membrane expression on neoplastic MCs compared to T lymphocytes. Although CD30 is not commonly expressed in normal marrows, when positive on the neoplastic MCs, it is usually weak to moderate, emphasizing the importance of proper optimization of immunohistochemistry by the laboratories to ensure their appropriate sensitivity. Assessment of the markers of aberrancy (i.e., CD2, CD25, CD30) on MCs via immunohistochemistry is much easier on serial section staining when the MC burden is high and dense MC aggregates are present. In small samples with few or no MC aggregates, serial sectioning may level through the area of interest. Interpretation of immunohistochemical staining in interstitial MCs or “occult” SM is much more challenging, requiring careful inspection of stained sections at higher magnification. In these circumstances, stains for CD25, CD117, and tryptase will be most useful, as these stains are typically strong and bright in SM [23].

Detection of mutated *KIT* as a minor diagnostic criterion for SM underscores the key pathogenic role of KIT signaling activation in SM. The canonical *KIT* p.D816V pathogenic variant is by far the most common molecular alteration in SM (over 90% of cases), but other mutations resulting in ligand‐independent KIT activation may occur [5]. *KIT* testing can be performed on peripheral blood, BM, or tissue samples. Because of the relatively low burden of the neoplastic MCs, molecular methodologies providing high sensitivity must be applied [24]. Currently, techniques approaching the sensitivity of 0.01%, such as highly sensitive digital droplet polymerase chain reaction (hs‐ddPCR) and highly sensitive allele‐specific oligonucleotide quantitative PCR (ASO‐qPCR), are becoming widely available. This testing, however, provides a *KIT* p.D816V‐centric approach. A reliably negative *KIT* p.D816V result by a highly sensitive analysis in an appropriate clinical setting should trigger additional molecular testing for an alternative *KIT* mutation at codon 816 and neighboring codons, followed by sequencing of the *KIT* gene [24]. Noteworthy is the lower sensitivity of next generation sequencing (NGS) and Sanger sequencing, which makes these techniques inadequate for the diagnosis of SM [25]. Recent data have shown new ultrasensitive molecular methods such as duplex sequencing and rolling circle amplification detecting *KIT* p.D816V in the blood of patients with indolent SM (ISM) down to 0.001% variant allele frequency (VAF) [26]. Despite the sensitivity of detection of the *KIT* p.D816V mutation in the peripheral blood of patients with SM, there is variability of its presence depending on the subtype, where ISM without skin lesions has a reported frequency of 66% detection as assessed by ASO qPCR [27]. Thus, the method of detection, sample source (e.g., blood vs. BM), and the exact diagnostic subtype of SM all affect the detection frequency of the *KIT* p.D816V mutation.

Despite the limited utility of NGS for the assessment of the *KIT* p.D816V mutation in SM [25], there remains an important role for NGS for myeloid mutations in all cases of SM at the time of initial BM examination [28]. As shown in Table 4, additional somatic variants are present far more frequently in advanced SM (AdvSM) compared to non‐advanced SM (NonAdvSM). The presence of these somatic variants may signal that an associated myeloid neoplasm (AMN) is present and/or represents an adverse prognostic marker of disease (e.g., the *SRSF2/ASXL1/RUNX1* genotype) [29]. Given the adverse prognostic effect of an AMN and the therapeutic decisions at risk, recognition of an SM‐AMN is critical. The pattern of the additional somatic variants present also provides clues to the type of AMN that could be present. Alternatively, the absence of additional somatic variants in the bone marrow of a patient with SM strongly argues for NonAdvSM. The presence of low‐level VAFs in *DNMT3A*, *TET2*, and/or *AXSL1* can signal the presence of clonal hematopoiesis of indeterminate potential (CHIP) or the so‐called clonal hematopoiesis of aging [30].

BST level is also used to diagnose SM [5]. BST represents mostly monomeric protryptases constitutively secreted by MCs, both normal and neoplastic. Increased BST, therefore, can serve as a surrogate marker for the overall MC burden and, as such, is commonly elevated in SM. Although BST of 20 ng/mL or higher is considered a diagnostic minor criterion, many patients with SM, especially ISM and BM mastocytosis, have BSTs below this threshold [31]. BST may also be elevated in many myeloid neoplasms that may or may not have a clonal MC involvement (e.g., acute myeloid leukemia, chronic myeloid leukemia, chronic myelomonocytic leukemia) [14]. Thus, BST should not be used as a SM criterion when an AMN is present. Additionally, a recently recognized condition characterized by a germline increased copy number of the *TPSAB1* gene encoding for α‐tryptase, also known as hereditary α‐tryptasemia (HαT), results in elevated BST levels [32]. The prevalence of HαT is enriched among patients with SM, occurring in approximately 15%–20% of individuals with SM versus 3%–8% in the general Western European population. In patients with confirmed HαT, BST levels can be adjusted by dividing the BST level by the number of extra *TPSAB1* genes +1 [32].

### 2.3 WHO Versus ICC Classification Systems

Several differences between the WHO 5th edition and ICC classifications should be noted (Table 2) [33]. When a major diagnostic criterion is present, the WHO requires at least one additional minor criterion to render a diagnosis of SM, whereas the ICC does not. The ICC specifically points out the importance of testing those patients with eosinophilia who lack *KIT* mutations (including canonical and alternative activating variants) for tyrosine kinase fusions associated with myeloid/lymphoid neoplasms with eosinophilia (M/LN‐eo) and tyrosine kinase gene fusions. The presence of such a fusion excludes SM even if large dense MC aggregates (major criterion) are present. Rare cases with a tyrosine kinase gene fusion and an activating *KIT* mutation should be reported as SM with associated M/LM‐eo.

**TABLE 2:** Major differences between diagnostic criteria for systemic mastocytosis by WHO, 5th edition (5) and ICC (7).

	WHO, 5th edition	ICC, 2022
Requirements for diagnosis	1 major and at least one minor criterion, or 3 minor criteria.	1 major criterion is sufficient, or 3 minor criteria.
Multifocal dense infiltrates of mast cells (major criterion)		Requires proper identification of mast cells by CD117 and/or tryptase immunopositivity.
*KIT* mutation (minor criterion)		In absence of *KIT* mutation, particularly in cases with eosinophilia, the presence of tyrosine kinase gene fusions must be excluded.
Basal serum tryptase (minor criterion), in the absence of an associated myeloid neoplasm	In cases with known hereditary α‐tryptasemia, tryptase levels should be adjusted.	

## 3 Subclassification of Systemic Mastocytosis

The multifactorial subclassification system for SM reflects the complexity and heterogeneity of the disease [34]. The drastic differences in prognosis, from normal to near‐normal life expectancy to highly aggressive forms of the disease, and treatment options necessitate the importance of correct SM subtyping [35, 36]. The subclassification of SM is based on the presence or absence of B‐findings (burden of disease) and C‐findings (cytoreduction‐required) (Table 3) [5]. SM is divided into non‐advanced and advanced categories mainly based on the overall prognosis. NonAdvSM includes ISM, BM mastocytosis, and smoldering SM. Aggressive SM, SM with associated hematologic/myeloid neoplasm (SM‐AHN/AMN), and MC leukemia (MCL) comprise AdvSM. BM mastocytosis has the overall best prognosis of any SM with disease confined to the BM, no skin lesions, no B‐ or C‐findings, and BST < 125 ng/mL. ISM typically has skin lesions, has 0–1 B‐findings, and no C‐findings, with a near‐normal life expectancy. Smoldering SM has at least 2 B‐findings, no C‐findings, and often has a high MC burden in the BM. Of the AdvSM, aggressive SM is defined by at least 1 C‐finding. SM‐AHN/AMN must meet diagnostic criteria of SM and an AHN/AMN. MCL has 20% or more MCs in BM aspirate or blood smears, and SM diagnostic criteria are fulfilled. Most *de novo* MCLs are “acute” with C‐findings being present, while “chronic” MCL, which lacks C‐findings, presents in fewer patients.

**TABLE 3:** B‐findings (“burden of disease”) and C‐findings (“cytoreduction‐required”) published by the World Health Organization, 5th edition (5) and International Consensus Classification (8).

	WHO, 5th edition	ICC, 2022
B‐findings	High mast cell burden – ≥ 30% mast cells in BM by histology (IHC) and/or – Serum tryptase ≥ 200 ng/mL and/or – *KIT* ^D816V^ VAF ≥ 10% in BM or PB leukocytes^a^	High mast cell burden – > 30% mast cells in BM and – Serum tryptase > 200 ng/mL
	Discrete and stable signs of myeloproliferation and/or myelodysplasia – Hypercellular BM and/or – Myelodysplasia (< 10% neutrophils, erythrocytes, and megakaryocytes)	Cytopenia (not meeting criteria for C findings) or ‐cytosis.^a^ Reactive causes are excluded, and criteria for other myeloid neoplasms are not met.
	Organomegaly – Hepatomegaly without ascites or other signs of organ damage or/and – Splenomegaly without hypersplenism and without weight loss or/and – Lymphadenopathy (palpable or visceral US‐ or CT‐found) (> 2 cm)^a^	Organomegaly – Hepatomegaly without impairment of liver function, or – Splenomegaly without features of hypersplenism including thrombocytopenia, and/or – Lymphadenopathy (> 1 cm size)^a^ on palpation or imaging
C‐findings	≥ 1 cytopenia – ANC < 1.0 × 10^9^/L – Hb < 10 g/dL, and/or – Platelet count < 100 × 10^9^/L	BM dysfunction caused by neoplastic mast cell infiltration manifested by ≥ 1 cytopenia – ANC < 1.0 × 10^9^/L – Hb < 10 g/dL, and/or – Platelet count < 100 × 10^9^/L
	Hepatopathy: ascites and elevated liver enzymes with/without hepatomegaly or cirrhotic liver with/without portal hypertension	Palpable hepatomegaly with impairment of liver function, ascites, and/or portal hypertension
	Palpable splenomegaly with hypersplenism with/without weight loss with/without hypoalbuminemia	Palpable splenomegaly with hypersplenism
	Malabsorption with hypoalbuminemia with/without weight loss	Malabsorption with weight loss due to gastrointestinal tract MC infiltrates
	Large‐sized osteolysis (≥ 20 mm) × with/without pathologic fracture with/without bone pain	Skeletal involvement with large osteolytic lesions^b^ with/without pathological fractures

Abbreviations: ANC, absolute neutrophil count; BM, bone marrow; CT, computer tomography; Hb, hemoglobin; IHC, immunohistochemistry; PB, peripheral blood; US, ultrasound; VAF, variant allele frequency.

^a^ Major differences between WHO, 5th edition and ICC, 2022 classification systems.

^b^ Although no specific size is indicated to qualify the osteolytic lesion as “large”, a size of ≥ 2 cm is used in clinical trials.

There are a few differences between the WHO 5th edition and the ICC for the subclassification of SM [33]. For example, BM mastocytosis is considered a separate subtype of SM by the WHO, but a clinicopathologic variant of ISM by the ICC. The ICC also restricts associated non‐MC neoplasms to myeloid neoplasms only (SM‐AMN) because a clonal relationship between the MC and non‐MC lymphoid component is exceedingly rare [6]. The ICC defines MCL more narrowly than the WHO 5th edition by restricting the diagnosis to cases with immature MCs; immature MCs can be identified on BM aspirate smears and touch preparations, or they may be seen in sheets in a BM biopsy [8].

## 4 Diagnostic Difficulties

The correct subtyping of SM requires integration of morphologic, laboratory, clinical, and imaging data [5]. There are clinicopathologic pearls that help in the diagnosis of SM variants (Table 4, Figure 1). Here, we will highlight the most common and clinically relevant diagnostic difficulties.

**TABLE 4:** Clues in the diagnosis of systemic mastocytosis variants.

	BMM	ISM	SSM	ASM	MCL	SM/MCL‐AHN/AMN
Blood						
MC%	0	0	0	0	Variable^a^	Variable^a^
Blast%	0	0	0	0	0	Variable^b^
Cytopenias^c^	No	No	No	Yes^c^	Variable^c^	Yes^c^
Cytoses	No	No	No	No	Variable^d^	Variable^d^
Bone marrow aspirate						
MC%	< 5	< 5	< 5	< 20	≥ 20	Variable
Myelodysplasia/myeloproliferation	No	No	Variable^e^	No	No	Yes
Bone marrow biopsy						
Cellularity away from MC aggregates	Normal	Normal	Hyper > Normal^e^	Normal	Normal	Hyper
MC%	< 5–10	< 10–20	> 30	> 50	> 50	Variable
Serum tryptase, ng/mL	< 125	≥ 20^f^	≥ 200	≥ 200	≥ 200	≥ 200
Abnormal karyotype	No	No	No	No	No	Yes
*KIT* D816V qPCR						
Blood^g^	Low/absent	Low	High	High	High	High
Bone marrow	Low	Low	High	High	High	High
Additional somatic mutations^h^	0	0–1^i^	0–1^i^	≥ 3	≥ 3	≥ 3

*Note*: Adapted from Table 1 [28].

Abbreviations: AHN/AMN, associated hematological neoplasm/associated myeloid neoplasm; ASM, aggressive systemic mastocytosis; BMM, bone marrow mastocytosis; hyper, hypercellular; ISM, indolent systemic mastocytosis; MC, mast cell; MCL, mast cell leukemia; qPCR, quantitative polymerase chain reaction; SSM, smoldering systemic mastocytosis.

^a^ Most MCLs are aleukemic as defined by < 10% circulating mast cells in blood; 10% or more circulating mast cells in blood is leukemic MCL.

^b^ Depending on the type of AHN, there may or may not be circulating blasts.

^c^ Cytopenias are commonly seen in ASM and acute MCL, as C‐findings (hemoglobin < 10 g/dL, ANC < 1.0 × 10^9^/L, platelets < 100 × 10^9^/L), but can also be seen secondary to an AHN. Chronic MCL, by definition, lacks C‐findings.

^d^ A mild increase in eosinophils (< 1.5 × 10^9^/L) may be seen in MCL. Other cytoses including eosinophilia > 1.5 × 10^9^/L can suggest an AHN. An absolute monocytes > 1.0 × 10^9^/L and > 10% monocytes on peripheral blood differential is suspicious for chronic myelomonocytic leukemia. Either an absolute monocytosis or > 10% monocytes, but not both, can suggest myelodysplastic/myeloproliferative neoplasm, unclassifiable.

^e^ Dysplasia or hypercellularity away from mast cell aggregates may be seen in SSM, but criteria are not met to diagnose an AHN/AMN per WHO or ICC criteria. Blood counts are normal or only slightly abnormal.

^f^ Serum tryptase levels are typically increased in ISM, but may be normal.

^g^ Discordantly high *KIT* D816V variant allele frequency in the blood when there is a low MC burden in the marrow is a surrogate for multilineage involvement/AHN.

^h^ Additional somatic mutations commonly include *SRSF2*, *ASXL1*, *RUNX1*, *CBL*, *JAK2*, and *EZH2*.

^i^ A subset (~25%) of ISM and SSM patients carries an additional mutation.

### 4.1 Mast Cell Leukemia

MCL, a very aggressive and rapidly fatal type of SM, may be difficult to recognize. The diagnosis requires a markedly increased number of MCs in the aspirate or blood smears (≥ 20%) (Figure 1) [34]. Unlike other leukemias, where neoplastic cells are readily seen in the peripheral blood, the majority of MCL cases are aleukemic (defined as < 10% circulating MCs); in practice, most MCLs have < 1% MCs in the peripheral blood smear [37]. Moreover, high MC burden in the BM biopsy is not diagnostic of MCL because patients with both NonAdvSM and AdvSM can demonstrate significant BM biopsy infiltration by neoplastic MCs. When accompanied by a high VAF of mutated *KIT* and/or high BST levels, a BM MC burden of ≥ 30% is considered a B‐finding (Table 3) and not indicative of MCL per se. The key diagnostic feature of MCL, ≥ 20% MCs in the aspirate smear, emphasizes the high diagnostic value of a well sampled BM aspirate and the ability of a morphologist to identify and enumerate MCs [38]. In MCL, more so than in other types of SM, MCs exhibit immature or pleomorphic morphology (e.g., promastocytes, metachromatic blasts, multinucleated, and highly atypical MC), sometimes with almost complete loss of cytoplasmic granules, which makes their recognition as MCs very difficult. In these situations, correlation of cell morphology between the aspirate smear and the core biopsy section stained with CD117 and tryptase antibodies may be beneficial. Other features, albeit non‐specific, may raise a suspicion of MCL, including a significant proportion of “promastocytes” in the touch preparations as well as a diffuse interstitial pattern of MC infiltration in BM core biopsy sections. As noted above, the ICC allows the use of diffuse marrow infiltration by immature atypical MCs (i.e., immature bilobed forms and multinucleated or bizarre MCs) to support a diagnosis of MCL in the proper clinical context when aspirate smears are suboptimal [8, 9, 10]. Another feature of MCL is a relative rarity of *KIT* p.D816V detected in approximately half of cases and alternative *KIT* mutations in MCL [37]. When diagnosed, MCL can be further subclassified into *de novo* or secondary, based on the absence or presence of a previous history of SM, respectively. WHO also supports distinctions between “acute” (detectable C‐findings) and “chronic” (absence of C‐findings) MCL as prognostically relevant [34].

### 4.2 Underrecognition of Associated Myeloid Neoplasms in Association With SM

The majority of advanced SM patients have SM with an AMN, where the latter is typically chronic myelomonocytic leukemia (Figure 1), chronic eosinophilic leukemia (CEL), or another myeloproliferative neoplasm (MPN), a myelodysplastic neoplasm (MDS), MDS/MPN‐U, or an acute myeloid leukemia (AML). The underrecognition of AMN can be addressed by the hematopathologist (Table 4). When SM is detected, the search should not stop there. Clues to the presence of an AHN/AMN may come from careful evaluation of peripheral blood and BM aspirate smears and the biopsy, supplemented with ancillary testing [16, 39]. Circulating blasts, cytopenias, and/or cytoses can all indicate an AMN. Dysplasia in all cell lineages, megakaryocytic atypia of an MPN, or mixed MDS/MPN suggest an AMN. Abnormal FC in non‐MC lineages also raises this possibility, as does an increase in CD34‐positive blasts by immunohistochemistry. Marrow hypercellularity away from dense MC infiltrates is often typical of an AMN. Although not always present in an AMN, an abnormal karyotype (other than loss of the Y chromosome) should raise a suspicion for SM‐AHN/AMN [40, 41]. The presence of non‐*KIT* mutations typically associated with AHNs further supports this possibility, as does a high *KIT* VAF in the peripheral blood in the absence of circulating MCs indicating the presence of the *KIT* mutation in an AHN/AMN and/or multilineage involvement [28].

### 4.3 Hidden Mastocytosis in Myeloid Malignancies

Certain myeloid malignancies, particularly AML with *RUNX1::RUNX1T1* fusion and chronic myelomonocytic leukemia, have a high association with SM [42]. In such cases, MC evaluation by CD117 and tryptase immunostains on BM biopsies will ensure the recognition of SM. Clinical clues, such as a lack of response to typical therapy in a patient with a myeloid neoplasm, should raise a suspicion for undiagnosed SM. For example, a patient with chronic myelomonocytic leukemia‐1 who does not respond to hypomethylating treatment and shows persistent organomegaly should undergo BM examination with a focus on potential MC disease. While BST levels can be elevated in myeloid malignancies, very high BST levels typically signal that SM is present. In our practice, we have encountered cases where SM was identified in myeloid malignancies with *KIT* p.D816V detected by NGS, similar to other institutions' experience [43, 44].

### 4.4 Recognition of B‐Findings and C‐Findings

Other challenges encompass the correct interpretation of morphologic and clinical data to document B‐ and C‐findings. The presence of one or more C‐findings defines aggressive SM, a SM subtype with poor prognosis. In the absence of C‐findings, two or more B‐findings would designate SM as smoldering SM. A morphologist often faces a challenge to interpret quantitative or qualitative abnormalities in non‐MC lineages. Before one considers cytoses and morphologic dysplasia as a B‐finding, or cytopenia(s) as a C‐finding, the possibility of an AMN must be considered and effectively ruled out. Otherwise, the case should be classified as SM‐AHN/AMN. The rest of the B‐ and C‐findings require clinical and laboratory information, often unavailable to a pathologist, so a multidisciplinary approach is key to correctly assess relevant abnormalities [45].

## 5 Morphologic Mimics

The morphologic mimics typically confused with advanced SM, especially SM‐AHN/AMN and MCL, include myeloid/lymphoid neoplasms with eosinophilia, myelomastocytic leukemia, and tryptase‐positive acute myeloid leukemia.

### 5.1 Myeloid/Lymphoid Neoplasms With Eosinophilia

In the differential diagnosis of SM‐AHN/AMN, one must consider a M/LN‐eo with recurrent genetic abnormalities [46], especially when a *KIT* mutation is not detected via a high sensitivity technique. M/LN‐eo with *PDGFRA*, *PDGFRB*, and *FGFR1* abnormalities all can have MC proliferations with aberrant CD25 expression and atypical spindle‐cell morphology; tryptase elevation is also a feature of CEL with *PDGFR* fusions. While the MC proliferations in M/LN‐eo are usually loose and thus dissimilar to the dense MC aggregates of SM, the distinction between the two diagnostic entities can be challenging [46]. In addition, some M/LN may present without eosinophilia [47]. An eosinophilia FISH or PCR panel and/or NGS panel with fusions is helpful in the situation where a MC proliferation is present, but an activating *KIT* mutation is lacking.

### 5.2 Myelomastocytic Leukemia

In the differential diagnosis of SM‐MDS or SM‐AML and MCL, is the very rare entity of myelomastocytic leukemia. This neoplasm, not recognized by the WHO and ICC, is composed of immature MCs (typically metachromatic blasts) and myeloblasts [48, 49]. Characterized by an aberrant karyotype, this disorder lacks diagnostic criteria for SM, including the *KIT* p.D816V, and does not respond to multikinase or selective tyrosine kinase inhibitors. Although up to 50% of MCL may lack a *KIT* mutation, most patients with MCL satisfy the basic SM diagnostic criteria. This is not true of myelomastocytic leukemia, which often resembles a high‐grade MDS with an immature MC proliferation.

### 5.3 Tryptase‐Positive Acute Myeloid Leukemia

When MCs are immature and round, such as in MCL and some cases of SM‐AHN/AMN, there is confusion between metachromatic blasts and myeloblasts. In rare cases of AML, myeloid blasts express tryptase, but in a typically dim and variable fashion [14]. BST is often also elevated, but the neoplastic cell morphology is that of usual myeloblasts. The distinctive large metachromatic granules seen in metachromatic blasts are lacking. Often complicating such cases are poor quality blood and aspirate smears, or inadequate specimen for FC. A complete AML work‐up will distinguish the tryptase‐positive AML from MCL or SM‐AHN/AMN.

## 6 Conclusions

Careful application of the diagnostic criteria for SM can lead to a correct diagnosis, but the work of the pathologist should not end there. Correlation with B‐ and C‐findings is necessary for the subclassification of SM as this drives therapy. Establishment of the presence or absence of *KIT* p.D816V using high‐sensitivity testing is essential. Most diagnostic difficulties occur with AdvSM rather than the relatively more frequent NonAdvSM. When one is evaluating aggressive SM, there should be a strong consideration of an AHN/AMN. Also, given the excellent response rates seen with selective tyrosine kinase inhibitors, failure to respond should trigger a re‐evaluation of the diagnosis of the patient.

## Author Contributions

Design of the work: T.I.G., A.R. Drafting of the work: T.I.G., A.R. Final approval of the version: T.I.G., A.R. Accountability for the work: T.I.G., A.R. Artificial Intelligence was not used for the generation of this manuscript.

## Ethics Statement

Ethics (IRB) approval is not necessary as this is a review article.

## Consent

The authors have nothing to report.

## Conflicts of Interest

T.I.G. has received consulting fees from Blueprint Medicines, Beckman Coulter, Cogent Biosciences, Celgene/BMS, and Incyte. A.R. has no conflicts of interest.

## Data Availability

Data sharing not applicable to this article as no datasets were generated or analyzed during the current study.
